# Impact of Dairy Products and Plant-Based Alternatives on Dental Health: Food Matrix Effects

**DOI:** 10.3390/nu15061469

**Published:** 2023-03-18

**Authors:** Blerina Shkembi, Thom Huppertz

**Affiliations:** 1 Food Quality & Design Group, Wageningen University & Research, 6708 WG Wageningen, The Netherlands; 2 FrieslandCampina, 3818 LE Amersfoort, The Netherlands

**Keywords:** carbohydrates, dairy products, dental health, dental caries, food matrix, minerals, protein, plant-based dairy alternatives

## Abstract

The impact of dairy products on dental health has been researched widely and shows an important role of various constituents, as well as the specific product matrix, in maintaining and improving dental health. These include, for instance, the position of lactose as the least cariogenic fermentable sugar, the high levels of calcium and phosphate, the presence of phosphopeptides as well as the antibacterial peptides lactoferrin and lysozyme and high buffering capacity. With plant-based alternatives for dairy products being developed and marketed these days, the specific benefits of dairy products in relation to dental health are often overlooked and most products contain more cariogenic carbohydrates, lack phosphopeptides, and have fewer minerals and less buffering capacity. Comparative studies performed to date indeed suggest that plant-based products do not match dairy counterparts when it comes to maintaining and improving dental health. Careful consideration of these aspects is required in relation to future developments of products and human diets. In this paper, we review the impact of dairy products and plant-based dairy alternatives on dental health.

## 1. Introduction

In recent years, a large number of plant-based products have been introduced into the market, particularly in Western countries, which are being positioned as dairy alternatives. While the main focus has been on liquid products positioned as alternatives for milk [1,2,3,4], acidified products positioned as yoghurt alternatives [5,6] and gelled products positioned as cheese alternatives [7,8,9] have also been introduced. One of the main focal points in the communication around the replacement of dairy products with these plant-based alternatives has been the environmental impact of the primary production of milk, which is often presumed higher than that of the plant-based raw materials used in the production of the aforementioned dairy alternatives. Such comparisons, however, may be relevant if one considers product replacement solely from a functional perspective, e.g., the whitening power of milk or plant-based products added to coffee or tea, but the comparisons do not consider nutritional equivalence. Recently, the Food and Agriculture Organization (FAO) stressed the importance of the consideration of (complimentary) functional units considering the nutritional value and/or health impact when conducting life cycle assessment (LCA) studies on food items [10]. If not considered, product replacement may impact overall nutritional and health status.

When considering the nutritional value and health impact of food products, a diverse range of metrics and indicators are available [11,12]. Comparison of products on the basis of a single nutrient may be straightforward but often leads to skewed comparison as hardly any food product’s contribution to the human diet can be adequately quantified on the basis of a single nutrient. When considering a broader spectrum of nutrients, various nutrient profiling scores are available, of which the nutrient-rich food (NRF) score is the most widely used [10]. Recent advances in nutrient profiling techniques also consider increased focus on those nutrients most at risk of deficiency in human diets [13]. However, nutrient profiling focuses primarily on nutrient sufficiency and often does not consider health impact beyond nutrient sufficiency. Dietary risk factors in relation to various non-communicable diseases (e.g., CVD, type-2 diabetes, or neoplasms) are considered in the Global Burden of Disease studies and consider wholefood effects related to e.g., the consumption of milk as well as legumes (but not of ingredients derived from these products) [14]. Recent advances by Stylianou et al. [15] also enable estimations of the dietary risk factors relating to aforementioned non-communicable diseases on a product basis. However, many potential impacts of diets and food items are not included in the aforementioned approaches, including, e.g., obesity, but also the impact of food items and their constituents on dental health.

The importance relation between dairy product consumption and dental health has been an important area of study for decades, and it has been established that dairy products provide protective effects [16,17,18]. This has been related to various aspects, e.g., the presence of high levels of calcium and phosphate in dairy products, the buffering capacity, the specific phosphorylated sequences in the main milk proteins, i.e., the caseins, as well as the fact that lactose is the least cariogenic of all dietary fermentable carbohydrates [16,17,18]. This combination of factors leads to the positive impact of the consumption of dairy products such as milk, cheese, and yoghurt on dental health. However, such beneficial effects for plant-based dairy alternatives may not be apparent due to the aforementioned differences in the composition of the products [1,2,3,4] but also differences in the specific components, e.g., in a type of protein or carbohydrate. In this paper, we review the impact of dairy products and plant-based dairy alternatives on dental health.

## 2. Dental Health and Foods

Good dental health is an essential part of overall health, well-being, and quality of life. Inadequate nutrition and poor oral hygiene can lead to dental diseases such as dental caries, enamel developmental defects, dental erosion, and periodontal disease (Table 1) [19,20,21,22,23,24,25,26,27]. Among these, dental caries is one of the prevalent dental chronic non-communicable diseases in the world, affecting people of all ages and strongly affected by food consumption [28]. Increased consumption of fermentable carbohydrates, mainly in the form of sugars found in foods and drinks, is significantly associated with an increased risk of dental caries [19,29]. When considering the effects of food products and their constituents on dental health, they are often categorised in terms of cariogenicity. Cariogenic refers to any substance that contributes to the development of caries and non-cariogenic applies to agents that do not promote or reduce dental caries [30]. The term cariostatic applies to substances that prevent or reduce the incidence of dental caries [30], whereas acidogenicity refers to the capacity to quickly catabolise simple carbohydrates such as sucrose, lactose, glucose, and fructose, producing organic acids as the main final product when these sugars are in excess [31].

Dental caries occurs because of the demineralisation of enamel and dentine [22,26], both of which consist primarily of calcium phosphate. Calcium phosphate salts are sparingly soluble in water at neutral pH, but solubility can be increased notably when pH is reduced [32,33,34]. When conditions are such that the liquid in contact with the dental surface is not saturated with calcium phosphate, calcium phosphate from the dental surface can solubilise, leading to the demineralisation of dental surfaces. The demineralisation process of enamel can typically occur at pH < 5.5–5.7, whereas for dentine it can already occur at pH < 6.2 [26]. Subsequently, it is the combination of pH and the duration of time that the surface is exposed to the pH that will determine the extent of demineralisation [35]. It should be noted that the aforementioned critical pH values apply to products not saturated in calcium phosphate. In a solution saturated with calcium phosphates at these pH values, such as e.g., milk or yoghurt, there is no driving force for the demineralisation of dental surfaces. Furthermore, it should be noted that saliva contains buffering compounds, i.e., protein, phosphates, and carbonates, so stimulation of saliva secretion can, at least partly, counter pH reductions in the mouth [36,37,38].

pH decreases in the oral cavity and on the dental surfaces can arise from two different routes, i.e., the consumption of acidic products and the in situ production of acids [22,26]. In situ production of organic acids occurs through the fermentation of carbohydrates, primarily by *Streptococcus mutans* [39]. Lactic acid is the main organic acid produced by this species, although in some cases some acetic and formic acid may also be produced [40]. For in situ acid production, the bacteria require fermentable sugars, which may be in the form of monosaccharides (e.g., fructose or glucose, but not galactose) and disaccharides (e.g., sucrose, maltose, and to a lesser extent lactose) that are present in the product or that can be formed from the breakdown of starch or starch derivatives via salivary amylase. However, consumption of fermentable sugars does not directly lead to dental caries; this also depends on various other factors such as dietary factors, food choices, salivary flow, fluoride exposure, and oral hygiene [22,41,42]. In addition, eating habits also have a great influence on the development of dental caries, such as, how often and fast we eat, or if we add sugar to our drinks [41,43]. Some key dietary factors and their effect on dental health are provided in Table 2. If not treated in time, dental caries can cause not only oral pain and tooth loss but also anxiety [22,28]. According to studies dental caries is the fourth most expensive disease to treat, consuming 5–10% of healthcare budgets in most industrialised countries, and is a common reason for hospitalisation of young children in some high-income countries [24,28,44]. Therefore, the promotion of proper nutrition education could be a strategy for reducing the risk of dental caries.

## 3. Influence of Consumption of Dairy Products and Plant-Based Alternatives on Dental Health

Plant-based products from soy, coconut, oat, hemp, rice, quinoa, almond, and other varieties of nuts are becoming increasingly popular as alternatives to bovine milk [1,74,75], however, scientific evidence points out their different cariogenic properties compared to bovine milk [76,77]. Dairy products such as milk, yoghurt, and cheese are typically considered non-cariogenic, which may be due to the high contents of calcium and phosphate, its high buffering capacity, lactose, casein, and casein phosphopeptides in dairy products [16,52,78], as described further in Section 4. It has also been suggested that lipids derived from dairy products may have a protective role, by creating a coating on enamel surfaces, thereby reducing the demineralisation of tooth enamel surfaces [56,69,70]. Lempert et al. [79] studied the association between the intake of dairy products and the development of dental caries in children 3–6 years old and found that a high intake of dairy products, as well as intakes of components of dairy such as dairy calcium, whey, and casein, was associated with less future caries development. In addition, Levy et al. [80] reported that higher consumption of milk at 24–36 months of age was related to a decreased risk of caries. Another study confirmed a negative association between dairy products and dental caries in 380 Greek adolescents 12–17 years old [81]. Petti et al. [82] investigated the relationship between milk and sucrose consumption and dental caries in 890 6–11-year-old Italian children and found that milk had a caries preventive effect only on those subjects with the highest frequency of sucrose consumption.

Johansson et al. [83] demonstrated that the prevalence of caries was lower in children who consumed milk with snacks compared to those who consumed snacks with other drinks (non-sweetened or sweet). In addition to milk, several studies have examined the relationship between cheese intake and dental caries. For example, Papas and colleagues [69] showed that increasing sugar consumption (more than two times per day) increased root caries, whereas increasing cheese consumption (more than two times per week) decreased the prevalence of root caries. It has been shown that cheese helps to restore plaque pH after sugar consumption, probably due to both effective saliva stimulation and the effect of its milk components [56]. In a study by Moynihan et al. [84] the consumption of cheese (15 g) by itself or as part of a cooked meal was found to increase the calcium concentration of dental plaque and thus may protect against dental caries. The protective effect of cheese intake against dental caries was also confirmed by Öhlund et al. [85].

Several mechanisms by which cheese may reduce cariogenicity have been suggested, i.e., (1) the stimulation of salivary flow, and the subsequent buffering effect of salivary compounds, neutralizing plaque acids, (2) inhibition of plaque bacteria reducing bacterial load and consequently reducing acid production [86]. Furthermore, cheese can also reduce cariogenicity through the release of high amounts of calcium and inorganic phosphate in dental plaque, which leads to a reduction in demineralisation and an enhancement in remineralisation [86]. Moreover, it has been shown that a high maternal intake of cheese during pregnancy was associated with a decreased risk of childhood dental caries [87].

Various studies have shown that the consumption of yoghurt may help to protect the gums and teeth from caries. For instance, Tanaka et al. [88] demonstrated that a high intake of yoghurt (4 times per week) was significantly associated with a lower prevalence of dental caries among Japanese children aged 3 years. In addition, Wu et al. [89] found that consuming yoghurt 2–4 times per week had a protective effect against dental caries in Chinese children aged 7–12 years. The protective effect of yoghurt and milk against dental caries was also confirmed by Petti et al. [90]. Furthermore, Ferrazzano et al. [53] demonstrated that the casein phosphopeptides in yoghurt were able to inhibit demineralisation and enhance dental enamel remineralisation. Another study by Ravishankar et al. [54] found in 68 voluntary students (34 with caries and 34 caries-free) aged 17–20 years that cheese and yoghurt without any added sugar (sucrose) were non-cariogenic as they increase calcium and phosphorus concentration and pH in dental plaque. A high concentration of calcium and phosphate ions in dental plaque may inhibit demineralisation and favour remineralisation of tooth enamel [54,56,71,86]. In order to reduce the prevalence of dental caries (especially in school-age children) the consumption of dairy products (without added sugar) as after-meal desserts have been recommended [54].

Wang et al. [91] found in American children and adolescents aged 2–17 years that high yoghurt intake (≥123 g/day) and low cheese intake (<22.6 g/day) were significantly associated with decreased risk of dental caries, as compared with non-consumers (reference group, intake = 0). Furthermore, it has been suggested that higher consumption of yoghurt products may protect also against tooth loss resulting from periodontal disease, probably modulating the oral microbiome composition [92]. The beneficial effect of dairy products against periodontitis has also been observed by several other investigators [93,94]. The anti-periodontitis activity of dairy products was attributed to calcium, which has been hypothesised to have an indirect effect on periodontal condition via the regulation of both skeletal and alveolar bone density [93,94,95].

From the preceding paragraphs, it is clear that there are numerous scientific studies on the influence of milk and dairy products on dental health, but comparatively few studies regarding the effects of plant-based dairy alternatives on dental health. Shen et al. [76] demonstrated in an in situ cross-over clinical study that the consumption of 200 mL of bovine milk resulted in remineralisation of the subsurface lesions of the enamel, whereas consumption of 200 mL of soy drink demineralised it, which may be attributable to the added sugar and the lower bioavailability of calcium in the soy drink. Guinot Jimeno et al. [96] evaluated the effect of plant-based beverages on the oral health of pediatric patients using scientific articles from PubMed published between December 2014 and November 2019 and found that plant-based drinks were more cariogenic than bovine milk; the authors suggested that this was not only due to the presence of added sugars in the plant-based drinks but also to their acidity. Dashper and colleagues [31] investigated the potential acidogenicity of soy and bovine milk beverages and found that soy drinks had a higher potential acidogenicity than bovine milk. Furthermore, soy beverages had a lower buffering capacity and a lower calcium bioavailability than those milk beverages, even though the total calcium content was similar, suggesting that may have a higher cariogenic potential than bovine milk beverages [31].

Lee et al. [75] estimated the cariogenic properties of soy beverage, whole bovine milk, and six different almond beverages, and analysed both their abilities to support *Streptococcus mutans* biofilm formation, acid production, and their capacity to buffer changes in pH. They found that the soy beverage supported the most biofilm growth and exhibited the highest cariogenic potential [75]. Widanti et al. [97] found that bovine milk was more effective for remineralising the enamel of demineralised teeth (caused by orange juice) than unsweetened soy drink, due to the higher amount of minerals for remineralisation in bovine milk compared to soy drink. Furthermore, Vongsavan et al. [98] demonstrated that soy drink enriched with calcium was not effective in protecting teeth against erosion induced by chlorinated water.

Using plaque pH measurement, a widely used method to study dietary cariogenicity, Danchaivijitr et al. [99] investigated the effect of different milk formulas (whole milk, soy-based, protein hydrolysate, and high and low casein ratio) on dental plaque pH and found that formulas containing only lactose as the sugar component caused significantly less plaque pH reduction than formulas contained other fermentable sugars (Supplementary Table S1). Levine [56] suggested that milk may reduce enamel demineralisation through three mechanisms: (1) milk proteins may get absorbed on the enamel surface and may prevent enamel demineralisation, (2) milk fat could adsorb on the enamel surface and may have a protective role, (3) milk enzymes present in milk may have a functional role in reducing the growth of acidogenic plaque bacteria. In the following section, the influence of specific food components present in milk and plant-based milk alternatives will be discussed further.

## 4. Influence of Components of Dairy Products and Plant-Based Alternatives on Dental Health

As outlined in Section 3, while beneficial effects on dairy health have been reported for several major classes of dairy products, e.g., milk, cheese, and yoghurt, such beneficial effects have not been reported for plant-based beverages positions as dairy alternatives. For plant-based products positioned as alternatives to yoghurt and cheese, no studies on dental health have been reported to date. When considering products in relation to nutrition and health, it is important to understand the role of specific constituents, and how they affect dental health, for dairy products and plant-based alternatives. The most important constituent groups in this respect are covered in this section. The manner in which they collectively interact, and create so-called matrix effects, is covered in Section 5.

### 4.1. Carbohydrates

When it comes to the relationship between food and dental health, carbohydrates are an important group of constituents to consider. While they are a major source of energy in the human diet, evidence also shows that dietary sugars and many other fermentable carbohydrates can be metabolised by oral microorganisms [26,27,45]. Fermentable carbohydrates include free sugars, glucose polymers, oligosaccharides, and highly refined starches and exclude non-starch polysaccharides and raw starches [100]. Free sugars refer to all monosaccharides and disaccharides that can be added to foods and beverages and sugars found naturally in fruit juices, syrups, honey, and fruit juice concentrates and are the most important risk factor for dental caries [19,28,44,101]. Brown sugars, fructose, sucrose, glucose, dextrose, and high-fructose corn syrup are some examples of added sugars [102]. Several studies suggested that the amount and the frequency of sugar consumption is an important factor for caries development [28,41,71,82,100]. Free sugars foods and/or drinks intake frequency must be restricted to a maximum of four times per day [100]. While sugars are naturally present in grains, whole fruits, vegetables, and dairy products do not play a significant role in the development of dental caries [28].

Carbohydrates are classified into three main categories: sugars, oligosaccharides, and polysaccharides. Sugars are classified as monosaccharides (glucose, fructose, and galactose) and disaccharides (sucrose, maltose, and lactose) [26,27,28]. Sugars can be readily metabolised by oral microorganisms involved in dental biofilm formation, producing organic acids, resulting in the demineralisation of the tooth structure [26,27,45]. Some studies reported a small difference between glucose, fructose, maltose, and sucrose reported in terms of acidogenicity [26,41]. In terms of cariogenicity, sucrose is considered the most cariogenic followed by glucose, maltose, and fructose [47,48]. Sucrose is considered the most cariogenic sugar, because, in addition to being fermented by oral microorganisms, it also serves as a substrate for the synthesis of extracellular and intracellular polysaccharides in dental plaque [49,50]. However, the cariogenicity of sucrose, and other sugars, is related to the amount and frequency of exposure [51]. Once per day exposure has been considered low consumption, three times per day moderate to high consumption, and five times or more high sucrose consumption [51]. The consumption of sugars as part of meals can reduce the risk of caries compared to when consumed between meals [26,27,103].

In contrast, lactose, the main carbohydrate in milk and most dairy products, has been shown to be less acidogenic than other sugars and less cariogenic [26,45,46]. Milk contains 4–5% of lactose, which can be fermented by oral microorganisms but the fermentation is significantly lower than from sucrose [16]. Johansson et al. [16] reported that sucrose reduced pH below 5.0, while lactose reduced it to around 6.0, and pH below 5.5 is considered dangerous for tooth enamel. Supplementary Table S1 shows the reported nutrient composition of various plant-based products and bovine milk taken from several studies published in the literature, as well as the USDA Food Central Database (https://fdc.nal.usda.gov/, accessed on 10 February 2023) [104]. Supplementary Table S1 shows that the total carbohydrate contents of the plant-based products range from 0.34 g/100 g in an almond drink to 10.3 g/100 g in a rice drink, and compared to bovine milk, where the only carbohydrate source is lactose, plant-based products contain sucrose, glucose, and maltose as the major types of carbohydrates, all of which are more cariogenic than lactose. Supplementary Table S1 also shows that most plant-based products with high sucrose concentrations are low in glucose and vice versa. Instead, the concentrations of fructose in most plant-based products are very low.

### 4.2. Minerals

In terms of the relationship between dietary mineral intake and dental health, dietary calcium and phosphorus, which are also the basic elements of hard dental tissues, have generated considerable interest [105]. Supplementary Table S1 shows that some plant-based drinks contain similar calcium and phosphate levels as bovine milk, though fortification, but that many products are notably lower in calcium content as well. However, Dashper et al. [31] found that even though the total calcium content was quite similar, the levels of soluble calcium in soy-based drinks were lower than in bovine milk beverages, which is likely due to the fact that calcium in plant-based drinks is in the form of added calcium phosphate or calcium carbonate, which are only sparingly soluble in water. Moreover, it has been shown that calcium in cheese or calcium and phosphates in milk and other dairy products help to restore minerals that the teeth may have lost due to other foods, thereby helping to rebuild tooth enamel [52]. Hence, dairy products increase calcium and phosphorus concentration in dental plaque, reducing demineralisation and enhancing remineralisation [52,53,54,55]. In addition, Lin et al. [106] found that the also dietary calcium and phosphorus ratio was strongly associated with caries development in human teeth. The protective role of calcium and phosphorus in milk and milk products against dental caries was also confirmed by Grenby et al. [107].

### 4.3. Proteins

In addition to lactose, calcium, and phosphorus, milk proteins have also been shown to affect dental health. Caseins are the most abundant group of proteins in bovine milk and accounts for approximately 80% of the total milk protein [57]. Reynolds and del Rio [58], and Reynolds and Black [59], reported that the addition of casein into drinking water and chocolate reduced dental caries in rats. Various mechanisms have been suggested regarding the anticaries action of casein, such as adsorption to the enamel, an increase of plaque pH by buffering acids, and also the presence of casein phosphopeptides [46,56]. Casein phosphopeptides (CPP), casein tryptic digestion products, have been shown to stabilise calcium phosphate in solution and to increase the level of calcium phosphate in dental plaque [60,61,62,63]. CPP, through their multiple phosphoseryl residues, can stabilise clusters of amorphous calcium phosphate (ACP) in metastable solution; preventing their growth to the critical size required for nucleation, phase transition, and precipitation [57,60,61,63,108]. It has been proposed that the mechanism of anticariogenicity of CPP-ACP is that they localise ACP in dental plaque, which buffers the free calcium and phosphate ion activities, helping to keep a state of supersaturation with respect to tooth enamel reducing demineralisation and promoting remineralisation [60,61,63]. In a study by Walker et al. [109] the remineralizing capacity of amorphous casein phosphopeptide (CPP-ACP) added to 200 mL of bovine milk in different amounts (2.0 and 5.0 g) was investigated. The authors found that enrichment of bovine milk with 2.0 or 5.0 g of amorphous casein phosphopeptide (CPP-ACP) significantly increased its ability in remineralizing enamel subsurface lesions in situ. An increase in mineral content was also found in CPP-ACP fortified milk compared to control milk (no CPP-ACP added) [109]. Moreover, Supplementary Table S1 shows that plant-based drinks, with the exception of soy drinks, are notably lower in total protein content compared to bovine milk. However, to date, little is known about plant proteins and their impact on dental health.

Other milk components such as lactoferrin, lysozyme, and lactoperoxidase may contribute to milk anti-cariogenicity, due to their antibacterial effect (Table 2) [16,57,64,65,66,67,68]. Lactoferrin is often used as an ingredient in oral many commercial products including toothpaste [68]. Lactoferrin for commercial use is primarily extracted from bovine milk [67]. Hatti et al. [65] found that a toothpaste containing lactoferrin together with lysozyme and lactoperoxidase prevents dental biofilm formation and exhibits antimicrobial properties when compared with a control toothpaste (fluoridated toothpaste, commercially available).

### 4.4. Trace Elements: Fluoride

Consuming foods containing fluoride has been found to reduce the incidence of dental caries, but excessive ingestion of fluoride can lead to dental fluorosis [41,71,72,73]. Fluoride ions play an important role in the protection against dental caries by inhibiting demineralisation and promoting remineralisation of tooth enamel [71,73]. However, it has been suggested that exposure to fluoride alone may not reduce caries but, together with reducing free sugars consumption, it has a significant effect on caries prevention [19]. Dashper et al. [31] shows that soy beverages contain a similar range of fluoride values (0.44–1.31 μg/mL) as bovine milk products (0.35–1.08 μg/mL) (Supplementary Table S2). Instead, a study conducted by Lal et al. [23] reported lower fluoride values of soy beverages (0.293 μg/mL, the median fluoride concentration of all products) (Supplementary Table S2). Lal et al. [23] also reported that the median (range) fluoride concentration of ultra-high temperature (UHT) soy beverages were lower than fresh soya beverages (0.272 μg/mL vs. 0.321 μg/mL), even though the number of products analyzed was different (42 vs. 10).

## 5. Dairy Products and Dental Health: Food Matrix Effects

From previous sections, it is clear that the components in milk and dairy products have unique properties when it comes to dental health. However, it is important to not only look at individual components, but also their interactions with the product, and how these affect overall outcomes. For instance, as recently reviewed, when it comes to glycemic responses of dairy products or dairy-based alternatives, it is not only the concentration and type of carbohydrate that is important but also all other macronutrients, as well as the structural organisation of the macronutrients, which is often governed by micronutrients such as calcium and other salts [110]. It is the overall structural organisation of the constituents of the product that governs the rate of release of carbohydrates from the stomach to the intestine, and therewith also the rate of absorption into the bloodstream and associated glycemic responses [110]. When it comes to dental health, similar matrix effects are apparent. In milk, for instance, it is the combination of carbohydrates with limited cariogenicity [16,17,18], i.e., lactose, that is present in a product of neutral pH and whose serum phase is saturated with respect to calcium phosphate [111] that results in an overall favourable response in relation to dental health. Such effects are further supported by the protein phase of milk, which contains the phosphopeptides embedded in the casein sequences [112] as well as some proteins with antimicrobial activity, e.g., lactoferrin and lysozyme, in relation to oral microbiota [16,57,65,67]. When milk is converted into yoghurt, pH decreases substantially, which could normally be considered a risk factor in relation to dental health. However, the natural role of the casein micelles in milk as encapsulants of large amounts of calcium phosphate, the so-called micellar calcium phosphate (MCP) [34,113,114] plays an important role here. Despite the acidification, the product still remains saturated in relation to calcium phosphate due to the fact that the MCP solubilises with decreasing pH [32,115,116]. Hence, despite the low pH, there is no notable driving force for the solubilisation of calcium phosphate from the enamel or dentine, due to the fact that the product remains saturated. Furthermore, it is interesting to note that the conversion of lactose to lactic acid by the starter bacteria in yoghurt manufacture mainly involved the glucose moieties of lactose, where the galactose moieties remain in the yoghurt [117]. For galactose, even anticaries effects have been reported based on the ability of galactose to occupy receptors of the pellicle and hence reduce or prevent adhesion of microorganisms [118]. Similar to yoghurt, cheese also has a notably lower pH than milk. However, with little or no residual carbohydrate in cheese, saturation with respect to calcium phosphate, and the stimulation of salivary secretion as a result of chewing [17,119] the semi-solid matrix also here a limited impact on dental health.

When it comes to plant-based products positioned as dairy alternatives, one can see that various protective mechanisms present in dairy products may be lacking. For instance, carbohydrate sources present in plant-based drinks (Supplementary Table S1) are typically more cariogenic than lactose, whereas buffering capacity is notably lower. Furthermore, calcium is typically added to these products in the form of calcium carbonate or calcium phosphate salts, which are sparingly soluble, have a tendency to sediment, and do not readily dissolve [1]. As such, they may not provide the same production against acidification as milk. Furthermore, the proteins in plant-based drinks do not naturally contain the phosphopeptide sequences which are found in caseins and some other animal food proteins [34]. As such, careful design of plant-based beverages is required to prevent the replacement of milk by these products does not cause negative effects on dental health. Likewise, when considering plant-based alternatives to yoghurt, similar points for consideration arise; again, such products often have a lower buffering capacity [120], may not be saturated with respect to calcium phosphate, and will contain more cariogenic carbohydrates than lactose and/or galactose. Furthermore, as for the drinks, the proteins used do not naturally embed. For (semi-hard) cheese alternatives, discrepancies are even larger, with many products currently available being starch-based rather than protein-based and containing much lower levels of calcium phosphate, if any [121]. Overall, it is thus apparent that while there will certainly be a place for plant-based products positioned as dairy alternatives in future diets, they may not readily mimic the properties of milk and dairy products when it comes to dental health. Careful consideration and product design appears warranted in this area.

## 6. Conclusions

When considering food products in relation to dental health, and particularly dental caries, dairy products milk, and dairy products take a special place. Their unique product matrices and the combination of constituents and their interactions creating this matrix ensure that they are not only a source of essential nutrients, but that these nutrients can also be provided with little or no negative impact on dental health, and can even positively impact dental health. Changing market trends towards the consumption of more plant-based dairy alternatives, require attention in this respect. Composition, components, and product matrices can differ strongly from their dairy counterparts, as a result of which dental health may be affected negatively, as evidenced by the recent. Research conducted to date indeed suggests such effects, related to e.g., compositional differences and different product matrices. These differences warrant considerable future research to ensure that potential effects on dental health are not overlooked in the development of plant-based products and their positioning as dairy alternatives. Such research should include both the evaluation of products on dental health as well as mechanistic studies on specific constituents present in the plant-based product matrices. Overall, the findings of this work highlight the necessity for critical evaluation of plant-based products as positioned dairy alternatives, which should go beyond nutrient content and composition.

## Figures and Tables

**Table 1:** Definitions of dental health diseases.

Dental Health Diseases	Definitions	References
Dental caries	is the demineralisation of dental hard tissues (enamel and dentine), caused by organic acids produced in the bacterial fermentation of dietary carbohydrates.	[19,22,26]
Periodontal disease	is a disease characterised by inflammation and damage of tooth-supporting structures.	[27]
Dental fluorosis	is a disease characterised by hypomineralisation of the tooth enamel, causing changes in the appearance of the enamel such as opaque spots or brown and yellow spots.	[23]
Dental erosion	is the irreversible loss of dental hard tissue caused by a chemical process without bacterial involvement.	[19,20]
Developmental Defects of Enamel	have been defined as disturbances in the matrix of hard tissues and their mineralisation during odontogenesis.	[25]

**Table 2:** The impact of dietary factors on dental health.

Dietary Factors	Dental Health Effect	References
Lactose	Limited cariogenicity	[26,45,46]
Sucrose	Cariogenic	[47,48,49,50,51]
Calcium	Protective	[52,53,54,55]
Phosphorus	Protective	[52,53,54,55]
Casein	Protective	[46,56,57,58,59,60,61,62,63]
Lactoferrin, lysozyme, and lactoperoxidase	Protective	[16,57,64,65,66,67,68]
Milk fat	Protective	[56,69,70]
Fluoride	Protective (not at a high amount)	[41,71,72,73]

## Data Availability

No new data were created or analyzed in this study. Data sharing is not applicable to this article.

## Supplementary Materials

The following supporting information can be downloaded at: https://www.mdpi.com/article/10.3390/nu15061469/s1, Table S1: Nutritional composition of plant-based drinks and bovine milk; Table S2: Fluoride concentration of plant-based drinks and bovine milk.
