# Supplementary material for: Impact of Dairy Products and Plant-Based Alternatives on Dental Health: Food Matrix Effects
Source: Nutrients. 2023 Mar 18;15(6):1469. doi: 10.3390/nu15061469 (PMC10056336; doi:10.3390/nu15061469)
Supplement: Supplementary file 1 [file nutrients-15-01469-s001.zip › nutrients-2250447-supplementary.pdf]

nutrients

MDPI

Table S1. Nutritional composition of plant-based drinks and bovine milk.

Type	Products name	Carbohydrate (g/100g)	Sugars (g/100g)	Sugar added	Glucose (g/100g)	Fructose (g/100g)	Sucrose (g/100g)	Maltose (g/100g)	Lactose (g/100g)	Calcium (mg/100g)	Phosphorus (mg/100g)	Protein (g/100g)	Fat (g/100g)	References
Bovine milk	Pura Milk Fresh	4.9 g/100 ml	4.9 g/100 ml	DNS						103.9 mg/100 ml	64.6 mg/100 ml	3.2 g/100 ml	3.4 g/100 ml	
Bovine milk	Pura Milk Long Life (UHT)	4.9 g/100 ml	4.9 g/100 ml	DNS						111.7 mg/100 ml	66.6 mg/100 ml	3.2 g/100 ml	3.4 g/100 ml	
Soy drink	So Good Lite	4.7 g/100 ml	1.9 g/100 ml	Corn maltodextrin, cane sugar	DNS	DNS	DNS	DNS	DNS	116.6 mg/100 ml	106.6 mg/100 ml	3.4 g/100 ml	0.9 g/100 ml	[31]
Soy drink	So Good Regular	5.0 g/100 ml	2.0 g/100 ml	Corn maltodextrin, cane sugar						119.7 mg/100 ml	89.6 mg/100 ml	3.4 g/100 ml	3.4 g/100 ml	
Soy drink	So Good Essential	6.3 g/100 ml	2.2 g/100 ml	Corn/wheat maltodextrin, cane sugar, corn syrup solids						131.2 mg/100 ml	102.2 mg/100 ml	3.2 g/100 ml	1.5 g/100 ml	
Soy drink	Vitasoy Original	6.1 g/100 ml	3.5 g/100 ml	DNS						127.9 mg/100 ml	76.6 mg/100 ml	3.3 g/100 ml	3.0 g/100 ml	
Follow-on formula—High casein	Bear 2			Lactose	DNS		DNS		20.52					
Follow-on formula—Low casein	Snow F Plus Beta			Lactose	DNS		DNS		2.6					
Follow-on formula—High casein	Meiji FU	DNS	DNS	Isomaltoligosaccharide, glucose, lactose	6.8	DNS	DNS	DNS	26.5	DNS	DNS	DNS	DNS	[99]
Follow-on formula—Low casein	Gain Advance			Sucrose, lactose	DNS		1.61		1.98					
Whole milk	Dumex 1 Plus			Lactose	DNS		DNS		5.01					
Whole milk	Honey Bear			Lactose, sucrose, maltodextrin, honey, oligo-fructose	DNS		10		2					
Whole milk	Dumex Dumilk			Sucrose	DNS		18		DNS					
Soy-based formula	Isomil			Sucrose, glucose syrup	DNS		10.4		DNS					
Protein hydrolysate-based formula	Pregestimil			Glucose syrup, maltodextrin	DNS		DNS		DNS					
Bovine milk	Woolworths Australian	4.8 g/100 ml	4.8 g/100 ml						4.8 g/100 ml	117 mg/100 ml		3.3 g/100 ml	3.4 g/100 ml	

Soy drink	Full Cream Milk Vitasoy® Milky Regular	3.1 g/100 ml	2.1 g/100 ml	DNS	DNS	DNS	DNS	DNS	DNS	120 mg/100 ml	DNS	3.0 g/100 ml	3.0 g/100 ml	[76]
Almond drink	Almond MLK		0.58		0.06	nd	0.52	nd				2.11	4.40	
Almond drink	Almond original		3.69		0.22	0.06	3.42	nd				0.41	1.18	
Almond drink	Organic almond drink		0.16		nd	nd	0.16	nd				0.95	3.69	
Almond drink	Carob almond MLK		4.58		0.87	0.61	3.10	nd				2.4	3.35	
Cashew drink	Organic cashew drink		2.87		0.49	1.96	0.43	nd				0.87	2.50	
Coconut drink	Coconut original		1.86		0.81	nd	nd	1.05				0.08	0.84	
Hazelnut drink	Hazelnut original	DNS	3.09	DNS	nd	nd	3.09	nd	DNS	DNS	DNS	0.36	1.56	[74]
Hemp drink	Hemp milk unsweetened		0.09		0.02	0.01	0.05	nd				0.08	2.44	
Macadamia drink	Organic macadamia drink		2.79		0.30	2.23	0.26	nd				0.29	2.62	
Oat drink	Organic oat drink		3.35		0.01	nd	nd	3.34				0.70	0.38	
Quinoa drink	Quinoa drink		3.2		0.43	2.34	nd	0.43				0.22	2.32	
Rice drink	Organic rice drink natural		7.02		4.12	0.07	nd	2.83				0.32	0.85	
Rice drink	Organic brown rice drink		5.58		3.07	0.10	nd	2.41				0.07	0.95	
Soy drink	Organic soya drink, calcium		2.43		0.50	1.27	nd	0.66				2.72	2.11	
Soy drink	Plain UHT organic soya drink		0.88		0.52	nd	0.36	nd				3.70	2.04	
Soy drink	Soya organic, wholebean		0.36		0.01	nd	0.35	nd				3.16	1.77	
Soy drink	Soya original		3.09		0.15	0.06	2.88	nd				2.61	1.48	
Bovine milk	Bovine fresh milk, pasteurised & homogenised		3.38		nd	nd	nd	nd				3.70	3.28	
Almond drink		1.3			0.01	0.001	1.25		DNS	65.6	43.4	1.02	2.56	
Cashew drink		2.28			0.03	0.0	0.36		DNS	6.4	33.7	1.33	2.76	
Coconut drink		2.77			0.31	0.0	1.6		DNS	47.1	29.6	0.32	1.02	
Bovine milk		5.03	DNS	DNS	0.01	0.0	0.0	DNS	5.02	112.1	92.4	3.26	3.54	[2]
Hemp drink		1.6			0.03	0.0	0.135		DNS	4.5	26.6	0.72	3.26	
Oat drink		3.67			3.32	0.09	0.15		DNS	49.9	28.9	0.46	1.45	
Rice drink		4.82			2.46	0.08	0.23		DNS	54.4	7.1	0.17	1.26	
Soy drink		1.47			0.01	0.001	1.4		DNS	84.2	80.7	3.78	2.06	
Spelt drink		4.95			3.72	0.11	0.07		DNS	12.1	31	0.72	1.22	
Soy drink		2.5		2.1 g/100g free sugar					0			3.2	2.0	
Oat drink		7.5		4.4 g/100g free sugar					0			0.8	1.3	
Almond drink		3.4	DNS	2.7 g/100g free sugar	DNS	DNS	DNS	DNS	0	DNS	DNS	0.6	2.2	[3]
Coconut drink		2.6		1.5 g/100g free sugar					0			0.1	1.5	

Rice drink		10.3		5.7 g/100g free sugar					0			0.2	0.9	
Semi-skimmed, bovine milk		4.8		0 g/100g free sugar					4.7			3.3	1.6	
Almond drink			<0.5		<0.1	<0.1	0.1	<0.1	<0.1	133	73	0.73	1.6	
Almond drink		DNS	<0.5	DNS	<0.1	<0.1	0.1	<0.1	<0.1	112	59	0.92	1.7	
Almond drink			<0.5		<0.1	<0.1	0.1	<0.1	<0.1	26	13.5	0.7	1.5	
Soy drink			1.7		<0.1	<0.1	1.6	0.1	<0.1	153	122	2.91	2.8	
Soy drink		DNS	2.1	DNS	<0.1	<0.1	2.1	<0.1	<0.1	17.3	39	2.8	2.6	
Soy drink			1.6		<0.1	<0.1	1.5	0.1	<0.1	129	110	2.91	2.7	
Coconut drink			2.1		<0.1	<0.1	2.1	<0.1	<0.1	125	61	0.44	2.3	
Coconut drink		DNS	<0.5	DNS	<0.1	<0.1	0.2	<0.1	<0.1	131	65	0.48	2.6	[1]
Coconut drink			3.1		<0.1	0.5	2.6	<0.1	<0.1	84	115	1.75	1.4	
Coconut drink			0.3		DNS	DNS	DNS	DNS	0	75	46	0.2	2.1	
Oat drink			1.8		0.2	<0.1	<0.1	1.6	<0.1	85	103	0.76	3	
Oat drink		DNS	1.1	DNS	0.3	<0.1	0.1	0.7	<0.1	132	118	0.67	2	
Oat drink			3.1		<0.1	<0.1	0.1	3	<0.1	65	32	1.11	1.3	
Oat drink			5.7		4.4	<0.1	0.1	0.9	0.2	3.7	12.1	0.64	1.3	
Rice drink			5.3		4.3	<0.1	0.1	0.9	<0.1	90	55	0.4	1.2	
Rice drink		DNS	4.1	DNS	0.3	<0.1	<0.1	3.8	<0.1	141	14.5	0.47	0.9	
Rice drink			4.3		0.3	<0.1	<0.1	4	<0.1	114	56	0.32	0.9	
Chilled milk			4.2		<0.1	<0.1	<0.1	<0.1	4.2	107	85	3.19	3.4	
Chilled milk		DNS	4.3	DNS	<0.1	<0.1	<0.1	<0.1	4.3	123	93	3.7	3.2	
Chilled milk			4.3		<0.1	<0.1	<0.1	<0.1	4.3	110	87	3.13	3.3	
UHT milk			4		<0.1	<0.1	<0.1	<0.1	4	116	94	3.45	3.4	
UHT milk		DNS	4.2	DNS	<0.1	<0.1	<0.1	<0.1	4.2	123	100	3.76	3.3	
UHT milk			4.1		<0.1	<0.1	<0.1	<0.1	4.1	114	92	3.64	3.2	
Bovine milk	Milk, whole, 3.25% milkfat	4.78	5.05	DNS	0	0	0	0	5.05	113	84	3.15	3.27	
Soy drink	Natural soy bean milk	2.63 g/100 ml	1.58 g/100 ml	DNS	DNS	DNS	DNS	DNS	DNS	0 mg/100 ml	DNS	3.16 g/100 ml	3.68 g/100 ml	
Soy drink	Original soy milk, original	3.75 g/100 ml	2.5 g/100 ml	DNS	DNS	DNS	DNS	DNS	DNS	188 mg/100 ml	104 mg/100 ml	3.33 g/100 ml	1.88 g/100 ml	
Soy drink	Organic edensoy original soy milk	5.83 g/100 ml	2.92 g/100 ml	DNS	DNS	DNS	DNS	DNS	DNS	42 mg/100 ml	62 mg/100 ml	4.58 g/100 ml	2.08 g/100 ml	
Soy drink	Soy milk, unsweetened, plain, refrigerated	3	DNS	DNS	<0.25	<0.25	2.58	<0.25	<0.25	155	46	2.78	1.96	
Soy drink	Soy milk, unsweetened, plain, shelf stable	1.29	0.56	DNS	<0.25	<0.25	0.56	<0.25	<0.25	101	69	3.55	2.12	
Almond drink	Almond milk, unsweetened, plain, refrigerated	0.67	DNS	DNS	<0.25	<0.25	0.04	<0.25	<0.25	158	19	0.66	1.56	
Almond drink	Almond milk, unsweetened, plain, shelf stable	0.34	0	DNS	<0.25	<0.25	<0.25	<0.25	<0.25	173	30	0.55	1.22	
Almond drink	Milked almonds	3.75 g/100 ml	2.92 g/100 ml	2.5 g/100 ml	DNS	DNS	DNS	DNS	DNS	3 mg/100 ml	DNS	2.08 g/100 ml	4. 58 g/100 ml	

Almond drink	Original almond milk, original	3.33 g/100 ml	2.92 g/100 ml	DNS	DNS	DNS	DNS	DNS	DNS	42 mg/100 ml	DNS	0.42 g/100 ml	1.04 g/100 ml	
Almond drink	Almond milk original	0.89	0	DNS	DNS	DNS	DNS	DNS	DNS	0	DNS	0.45	1.56	
Oat drink	Milked oats	7.5 g/100 ml	2.08 g/100 ml	1.7 g/100 ml	DNS	DNS	DNS	DNS	DNS	8 mg/100 ml	DNS	1.67 g/100 ml	0.62 g/100 ml	[104]
Oat drink	The original oat-milk	6.67 g/100 ml	2.92 g/100 ml	DNS	DNS	DNS	DNS	DNS	DNS	146 mg/100 ml	112 mg/100 ml	1.25 g/100 ml	2.08 g/100 ml	
Oat drink	Original milked oats, original	7.32 g/100 ml	1.97 g/100 ml	1.4 g/100 ml	DNS	DNS	DNS	DNS	DNS	8 mg/100 ml	DNS	1.13 g/100 ml	0.7 g/100 ml	
Oat drink	Original milked oats, original	7.38 g/100 ml	1.85 g/100 ml	1.5 g/100 ml	DNS	DNS	DNS	DNS	DNS	8 mg/100 ml	DNS	1.23 g/100 ml	0.77 g/100 ml	
Oat drink	Organic barista oat milk	9.3 g/100 ml	4.65 g/100 ml	DNS	DNS	DNS	DNS	DNS	DNS	0 mg/100 ml	DNS	0.42 g/100 ml	2.11 g/100 ml	
Oat drink	The original oat-milk, original	6.67 g/100 ml	3.03 g/100 ml	DNS	DNS	DNS	DNS	DNS	DNS	106 mg/100 ml	91 mg/100 ml	1.21 g/100 ml	2.12 g/100 ml	
Oat drink	Original organic oat milk, original	7.08 g/100 ml	2.5 g/100 ml	2.5 g/100 ml	DNS	DNS	DNS	DNS	DNS	4 mg/100 ml	DNS	1.25 g/100 ml	0.62 g/100 ml	
Oat drink	Original plant-based oat milk, original	8.75 g/100 ml	5.42 g/100 ml	DNS	DNS	DNS	DNS	DNS	DNS	121 mg/100 ml	DNS	0.83 g/100 ml	1.67 g/100 ml	
Oat drink	Unsweetened milked oats, unsweetened	5.83 g/100 ml	0.42 g/100 ml	DNS	DNS	DNS	DNS	DNS	DNS	8 mg/100 ml	DNS	1.67 g/100 ml	0.62 g/100 ml	
Oat drink	Oat milk, unsweetened, plain, refrigerated	5.1	2.32	DNS	0.77	<0.25	<0.25	1.55	<0.25	148	89	0.8	2.75	
Nut & oat drink	Organic nut & oat milks blend	2.95 g/100 ml	0.84 g/100 ml	DNS	DNS	DNS	DNS	DNS	DNS	8 mg/100 ml	DNS	1.69 g/100 ml	3.38 g/100 ml	
Nut & oat drink	Organic nut & oat milks blend	3.38 g/100 ml	1.27 g/100 ml	DNS	DNS	DNS	DNS	DNS	DNS	17 mg/100 ml	DNS	1.69 g/100 ml	4.64 g/100 ml	
Rice drink	Rice drink, original	9.58 g/100 ml	4.17 g/100 ml	DNS	DNS	DNS	DNS	DNS	DNS	125 mg/100 ml	62 mg/100 ml	0.42 g/100 ml	1.04 g/100 ml	
Rice drink	Original organic non-dairy beverage rice drink, original	9.17 g/100 ml	2.5 g/100 ml	DNS	DNS	DNS	DNS	DNS	DNS	146 mg/100 ml	DNS	0.42 g/100 ml	1.04 g/100 ml	
Rice drink	Organic sprouted rice drink, original	9.58 g/100 ml	4.17 g/100 ml	DNS	DNS	DNS	DNS	DNS	DNS	125 mg/100 ml	62 mg/100 ml	0.42 g/100 ml	1.04 g/100 ml	
Rice & quinoa drink	Dream blends, unsweetened enriched rice & quinoa drink, original, original	3.75 g/100 ml	0.42 g/100 ml	DNS	DNS	DNS	DNS	DNS	DNS	125 mg/100 ml	DNS	0.42 g/100 ml	1.04 g/100 ml	
Almond drink*	Silk Original Almond		2.92 g/100 ml	cane sugar								0.42 g/100 ml		
Almond drink*	Silk Original Unsweetened Almond		0 g/100 ml	none								0.42 g/100 ml		

Almond drink*	Silk Vanilla Almond		6.67 g/100 ml	cane sugar								0.42 g/100 ml		
Almond drink*	Silk Chocolate Almond	DNS	7.08 g/100 ml	cane sugar cocoa	DNS	DNS	DNS	DNS	DNS	DNS	DNS	0.42 g/100 ml	DNS	[75]
Almond drink*	Almond Breeze Original Almond		2.92 g/100 ml	evaporated cane juice								0.42 g/100 ml		
Almond drink*	Almond Breeze Original Unsweetened Almond		0 g/100 ml	none								0.42 g/100 ml		
Bovine milk*	Horizon Whole Milk		4.58 g/100 ml	none								3.33 g/100 ml		
Soy drink*	Silk Soy Milk		2.5 g/100 ml	none								3.33 g/100 ml		

*Values for products calculated assuming a serving size of 240 mL; DNS: refers to data non supplied; nd: refers to not detectable.

Table S2. Fluoride concentration of plant based drinks and bovine milk.

Type	Products Name	Fluoride (μg/mL or ppm)	References
Bovine milk	Pura Milk Fresh	0.35	[31]
Bovine milk	Pura Milk Long Life (UHT)	1.08	
Soy drink	So Good Lite	0.67	
Soy drink	So Good Regular	1.31	
Soy drink	So Good Essential	0.51	
Soy drink	Vitasoy Original	0.44	
Soy drink	Asda	0.065	[23]
Soy drink	Alpro	0.603	
Soy drink	Calsoy	0.015	
Soy drink	Cooperative	0.250	
Soy drink	Holland and Barrett	0.231	
Soy drink	Marks and Spencer	0.261	
Soy drink	Morrison	0.322	
Soy drink	Provamel	0.327	
Soy drink	Sainsbury	0.274	
Soy drink	Soya Soleil	0.066	
Soy drink	Tesco	0.279	
Soy drink	Vitasoy	0.376	
Soy drink	Vivesoy	0.391	
Soy drink	Waitrose	0.224	
Soy drink	Yeo	0.381	
